# RNF135, RING finger protein, promotes the proliferation of human glioblastoma cells *in vivo* and *in vitro* via the ERK pathway

**DOI:** 10.1038/srep20642

**Published:** 2016-02-09

**Authors:** Yongjian Liu, Feng Wang, Yongsheng Liu, Yiqun Yao, Xiupeng Lv, Bin Dong, Jun Li, Siyang Ren, Yiwen Yao, Yinghui Xu

**Affiliations:** 1 Department of Interventional therapy, First Affiliated Hospital, Dalian Medical University, Dalian, People’s Republic of China; 2 Department of Neurosurgery, First Affiliated Hospital, Dalian Medical University, Dalian, People’s Republic of China

## Abstract

Ring finger protein 135 (RNF135), located on chromosome 17q11.2, is a RING finger domain-containing E3 ubiquitin ligase that was identified as a bio-marker and therapy target of glioblastoma. In our study, we confirmed that RNF135 was up-regulated in glioblastoma tissues compared with normal brain (NB) tissues, and that RNF135 knockdown inhibited proliferation and migration and led to cell cycle arrest in the G0/G1 phase *in vivo*. By lowering RNF135 expression, phosphorylated Erk and cell cycle protein CDK4 were down-regulated, while p27^Kip1^ and p21^Waf1/Cip1^ were up-regulated in U87 and U251 cells *in vitro*. In addition, using the immunofluorescence double labelling method, we found that RNF135 and P-Erk were co-localized in the cytoplasm and were highly expressed in glioblastoma samples compared with NB tissues. Moreover, the growth of U87 cell-transplanted tumours in nude mice was inhibited while transduced with Lv-shRNF135. Taken together, our findings demonstrate the biological effects of RNF135 in glioblastoma cell proliferation, migration and cell cycle, and its role in the progression of glioblastoma may be associated with the ERK signal transduction pathway.

Glioblastoma multiforme (GBM) is regarded as one of the most common tumours in the central nervous system in adults and is associated with an unfavourable outcome. Despite the great advances in therapeutic strategies, including surgery, chemotherapy and radiotherapy, the median survival of GBM patients is still a mere fourteen months^1^,^2^. Glioblastoma is considered the most malignant, aggressive and lethal glioma^3^. The WHO classification system provides information on prognosis and management of malignant gliomas^4^. With the advances in molecular biology technologies, an increasing number of biomarkers and therapeutic targets have been identified, including TP53 mutations, loss of heterozygosity (LOH) 10q, PTEN mutations, EGFR amplification and E3 ubiquitin ligases^5^,^6^,^7^.

E3 ubiquitin ligases, a large family of proteins, regulate the turnover and activity of many target proteins. Previous studies have revealed that the E3 activity of Mdm2 towards p53 is due to its atypical C-terminal RING finger domain^8^,^9^. The RING fingers, defined by the sequence CX2CX (9–39) CX (1–3) HX (2–3) C/HX2CX (4–48), are categorized as RING-HC and RING-H2^10^. RING fingers with E3s play key roles in various cellular processes and may contribute to disease. RING finger E3s influence the balance between cell proliferation and apoptosis. The E3 activity of IAPs (RING finger-containing inhibitors of apoptosis) results in their ubiquitination, degradation, and progression towards cell death when exposed to apoptotic stimuli^11^. A growing body of evidence has suggested that E3 ubiquitin ligases are involved in cancer development and are overexpressed in many human cancers^12^,^13^.

Ring finger proteins are RING finger domain-containing E3 ubiquitin ligases involved in the regulation of gene transcription, translation, cell adhesion, and epithelial development^14^. RNF135 protein consists of an N-terminal RING finger domain and C-terminal SPRY and PRY motifs^15^, and belongs to the E3 ubiquitin ligase family. It has been reported that RNF135 ubiquitinates RIG-I (retinoic acid-inducible gene-I) and promotes its signal transduction capacity to produce antiviral IFN (type-I interferon)^15^,^16^,^17^,^18^. Moreover, the role of RNF135 in cancer was also reported previously. The RNF135 gene was demonstrated to be down-regulated in Schwann tumour cells from malignant peripheral nerve sheath tumours, and it was also suggested that RNF135 was involved in the increased malignant risk in NF1 micro deletion patients^19^. However, the function and expression levels of the RNF135 gene in human glioblastoma remain unknown.

In the present study, we confirmed that RNF135 expression was increased in human patient samples. A lentivirus-mediated RNA interference (RNAi) system was used to explore the effect of the RNF135 gene on the migration, proliferation and cell cycles of U87 and U251 cells *in vitro*. Furthermore, the possible mechanisms involved in this process were also investigated. Our findings provide new insights into the role of RNF135 in the development of glioblastoma, and implicate the potential application of RNF135 in glioblastoma therapy.

## Results

### RNF135 is highly expressed in glioblastoma tissues compared with NB tissues

**Figure 1:**
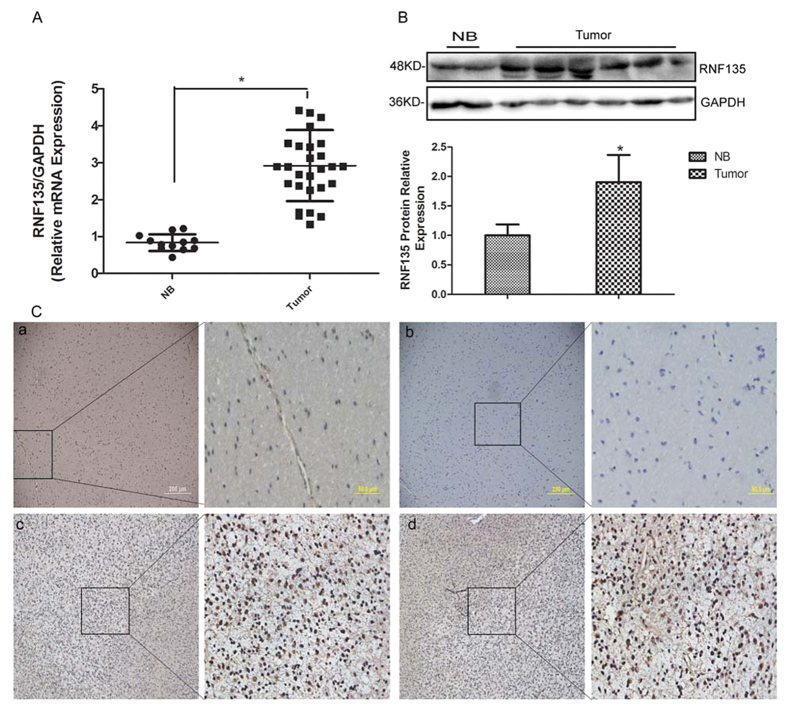
RNF135 is highly expressed in human malignant glioma tissues (tumour) compared with normal brain tissues (NB). (**A**) The mRNA expression of RNF135 is increased in tumour tissues (28 clinical cases) compared with NB tissues (12 clinical cases) by Q-RT-PCR assay. GAPDH was used as a loading control. (**B**) Western blot assay was used to test the RNF135 protein expression levels between NB tissues (12 clinical cases) and glioblastoma tissues (28 clinical cases) with GAPDH as the loading control. Data are presented as the mean ± SD for three independent experiments. (**C**) Representative IHC images showed RNF135 expression was decreased in 15 normal brain tissues (**a,b**) compared with 142 primary glioma samples (**c,d**). (Original magnification: 400×,100×).

To evaluate the role of RNF135 in glioblastoma, quantitative real-time PCR and Western blotting were employed to detect the expression of RNF135 in 28 fresh glioblastoma samples and 12 NB tissues. The data of quantitative real-time PCR showed that glioblastoma tissues presented much higher transcription levels of RNF135 than NB tissues (P < 0.05) (Fig. 1A). Similarly, the RNF135 protein levels in glioblastoma tissues were also higher than those in NB tissues (Fig. 1B) (P < 0.05). Furthermore, to measure the expression levels of RNF135 in different pathological grade gliomas and normal brain tissues, we examined the RNF135 expression levels in 154 archived paraffin-embedded samples, including 14 astrocytic tumours, 17 oligodendroglioma, 66 anaplastic astrocytoma and 45 glioblastoma and 12 NB tissues, using the immunohistochemical (IHC) staining assay (Fig. 1C). The IHC assay demonstrated that the RNF135 protein was highly expressed in approximately 66.9% of glioma tissues but only highly expressed in 25% of NB tissues (P = 0.009) (Table 1).

### Relationship between RNF135 expression and clinicopathologic characteristics in glioma patients

The clinicopathologic characteristics of the patients are shown in Table 2. There was no remarkable association between RNF135 expression and the patients’ age and gender in the randomly selected glioma cases. Nevertheless, the levels of RNF135 expression were correlated with the grade of pathology classification (WHO I–II vs WHO III–IV) and histologic type in glioma patients (P = 0.000). In addition, to confirm whether the RNF135 expression levels and some other clinical factors were the independent factors of prognostic prediction in glioma patients, we performed univariate and multivariate analyses using the Cox proportional hazard regression model. The results showed that the RNF135 expression level (P = 0.000; HR: 2.958; multivariate Cox regression analysis) and the radiochemotherapy (p = 0.02; HR: 0.573; multivariate Cox regression analysis) both were indicative of significant prognostic factors of glioma in patient survival time and the RNF135 expression levels was a more important factor (Table 3).

### High expression of RNF135 predicts a negative survival in glioma patients

**Figure 2:**
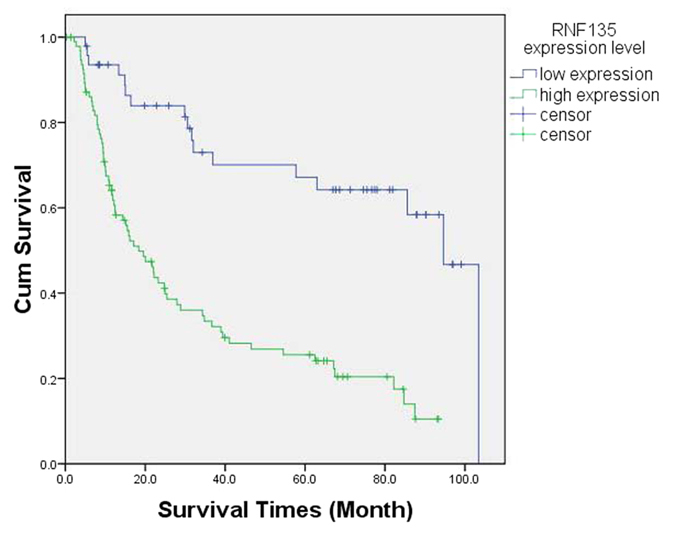
Kaplan-Meier survival analysis of overall survival duration in 142 glioma patients according to RNF135 protein expression. Over-expression of RNF135 was a negative factor for prognosis of glioma. The log-rank test was used to calculate P values. (high expression: high-express of RNF135; low expression: low-expression of RNF135).

To explore the prognostic value of RNF135 expression in glioma, the relationship between RNF135 expression levels and 142 glioma patients’ survival was assessed by Kaplan-Meier analysis and log-rank test. Low levels of RNF135 expression in glioma tissues correlated significantly with a better survival than those with high-expression (P = 0.000, log-rank test) (Fig. 2).

### Successful establishment of steadily down-regulated RNF135 expression in U87 and U251 cell lines

**Figure 3:**
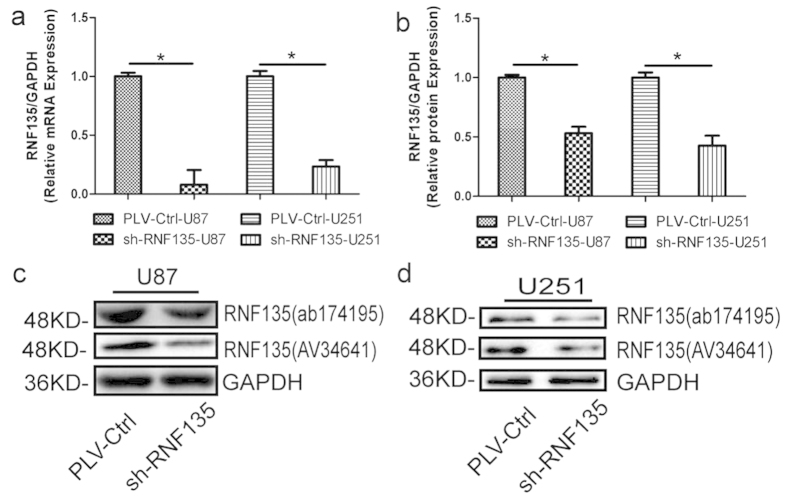
ShRNA stably down-regulates RNF135 expression in the human glioma cell lines U87 and U251. (**a**) Q-RT-PCR assay shows the transcriptional levels of the RNF135 gene with GAPDH used as the loading control. (**b–d**) Western blot assay shows that the RNF135 protein expression levels in sh-RNF135 and PLV-Ctrl treatments. GAPDH served as the loading control. Data are presented as the mean ± SD for three independent experiments (P < 0.05).

To determine the effect of RNF135, the lentiviral shRNA vector was used to stably and specifically lower RNF135 expression in U87 and U251 cells established from high-grade tumours. The RNF135 mRNA and protein expression levels were measured by quantitative real-time PCR and Western blotting. Respectively, the RNF135 mRNA (Fig. 3a,b) and protein expression levels (Fig. 3c,d) were significantly attenuated compared with the negative vector control [pLVTHMGFP-Control (PLV-Ctr)] (P < 0.05).

### Stably down-regulated RNF135 expression suppresses cell proliferation and migration *in vitro*

**Figure 4:**
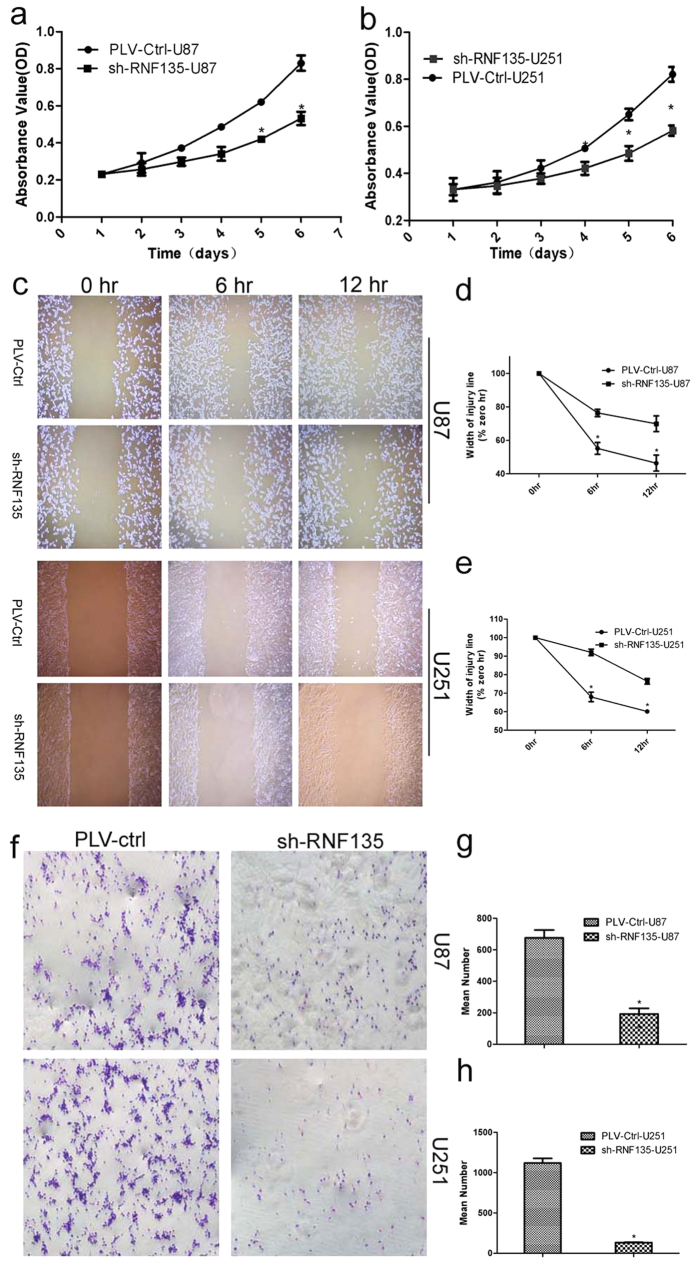
Stable knockdown of RNF135 suppresses cell proliferation and migration *in vitro*. (**a,b**) The MTT assay was used to measure the proliferation of U87 and U251 cells down-regulated by shRNF135. Absorbance was read at 490 nm using averages from triplicate wells. (**c–e**) Stable knockdown of RNF135 reduced the migration ability of U87 and U251 cell lines *in vitro*. (**f–h**) The Boyden chamber assay was used to measure the migration effect. Data are presented as the mean ± SD for three independent experiments (*P < 0.05, statistically significant difference) (Original magnification: 100×).

To investigate the biological implication of RNF135 cells in glioblastoma, we conducted MTT, wound healing and Boyden chamber analysis experiments to determine the effect of decreased RNF135 expression on glioblastoma cell growth and migration *in vitro*. We performed the MTT assay to measure the effect of RNF135 on U87 and U251 cell line growth and proliferation. The results from this assay showed that cells transfected with shRNF135 grew significantly slower than shPLV-Ctrl cells (P < 0.05) (Fig. 4a,b). Next, to examine the effect of RNF135 on glioblastoma cell migration, we performed wound healing and Boyden chamber assays. The results of the wound healing assay showed that shRNF135 U87 and U251 cells presented a lower speed into the gap than PLV-Ctrl cells (P < 0.05) (Fig. 3c–e).The Boyden chamber assay revealed fewer U87 and U251 cells on the lower surface than PLV-Ctrl cells. The strained cells were counted using Image J software (P < 0.05) (Fig. 4f–h). The results inferred that knockdown of RNF135 expression could reduce the migration effect on U87 and U251 cells.

### Knockdown of RNF135 could arrest the cell cycle in the G0/G1 phase in U87 and U251 cells

**Figure 5:**
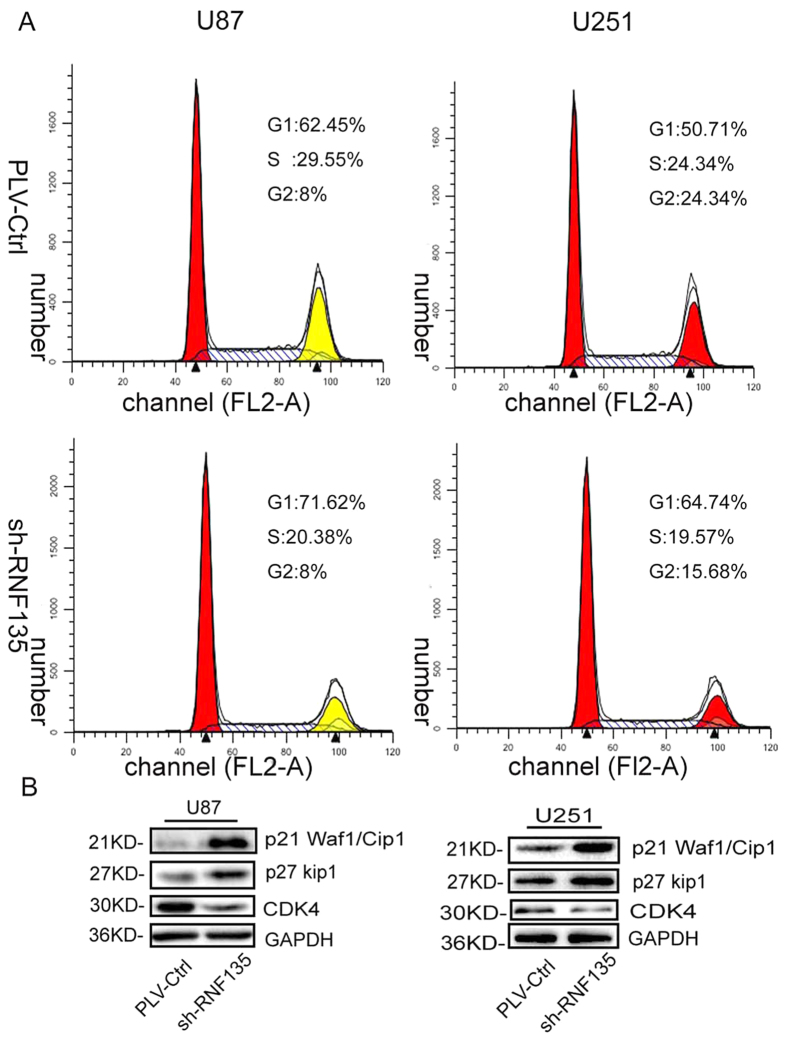
Down-regulated RNF135 expression arrested cell cycle at G0/G1 phase and regulated CDK4, p27 and p21 expression in U87 and U251 cells *in vitro*. (**A**) Effect of the influence of glioblastoma cells by transfected with sh-RNF135 on cell cycle progression. The cell cycle distribution of U87 cells transfected with sh-RNF135 and PLV-Ctrl were measured using propidium iodide staining and flow cytometry analyses. (**B**) Knocking down endogenous RNF135 expression reduced the expression of CDK4. However, p21 and p27 were highly expressed. GAPDH was used as a loading control. Data are presented for three independent experiments.

**Figure 6:**
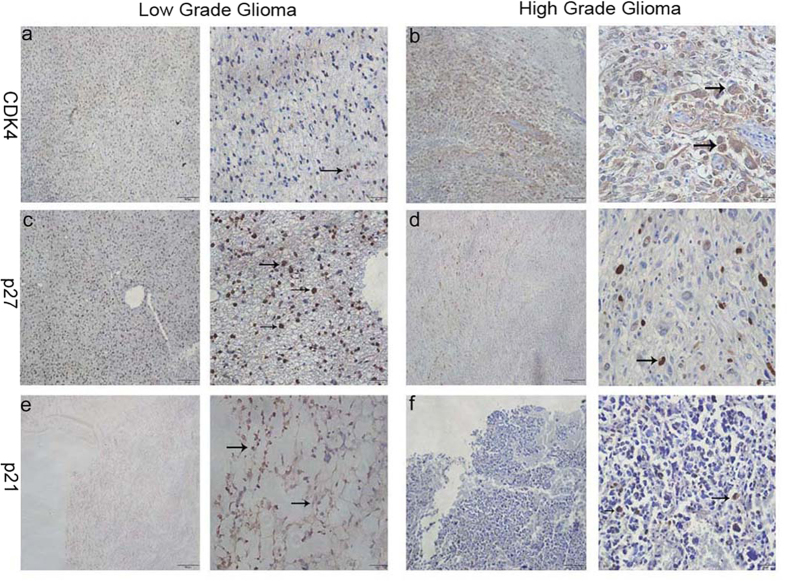
Expression of CDK4, p27 and p21 in low grade and high grade glioma tissues. (**a,b**) The IHC assay show the CDK4 was high expressed in high grade glioma compared with low grade glioma (arrow). (**c–f**) the p27 and p21 displayed the opposite result (arrow) (Original magnification: 400×, 100×).

We explored the effect of low expression of RNF135 on cell cycle distribution in U87 and U251 cells by flow cytometry plus propidium iodide (PI) staining. The results revealed 71.62% and 64.74% of G0/G1 phase cells in the shRNF135 U87 and U251 cells, respectively, and 62.45% and 50.71% of G0/G1 phase cells in the PLV-Ctr cells, respectively (P < 0.05) (Fig. 5A). These data indicate that the cell cycle was arrested in the G0/G1 phase after knockdown of RNF135 expression in U87 and U251 cells. Moreover, the expression levels of cell cycle regulation proteins p27^Kip1^, p21^Waf1/Cip1^ and CDK4 were determined using Western blot analysis (Fig. 5B). Our results suggested that low RNF135 expression could reduce CDK4 expression, while enhancing the expression of p27^Kip1^ and p21^Waf1/Cip1^. To measure the expression of p27^Kip1^, p21^Waf1/Cip1^ and CDK4 in glioma tissues, we performed the IHC staining assay (Fig. 6). In this assay, we found that CDK4 was highly expressed in high-grade glioma tissues compared with low-grade glioma tissues (Fig. 6a,b); however, p27^Kip1^ and p21^Waf1/Cip1^ showed opposite results (Fig. 6c–f).

### Knockdown of RNF135 could attenuate the activation of p-Erk

**Figure 7:**
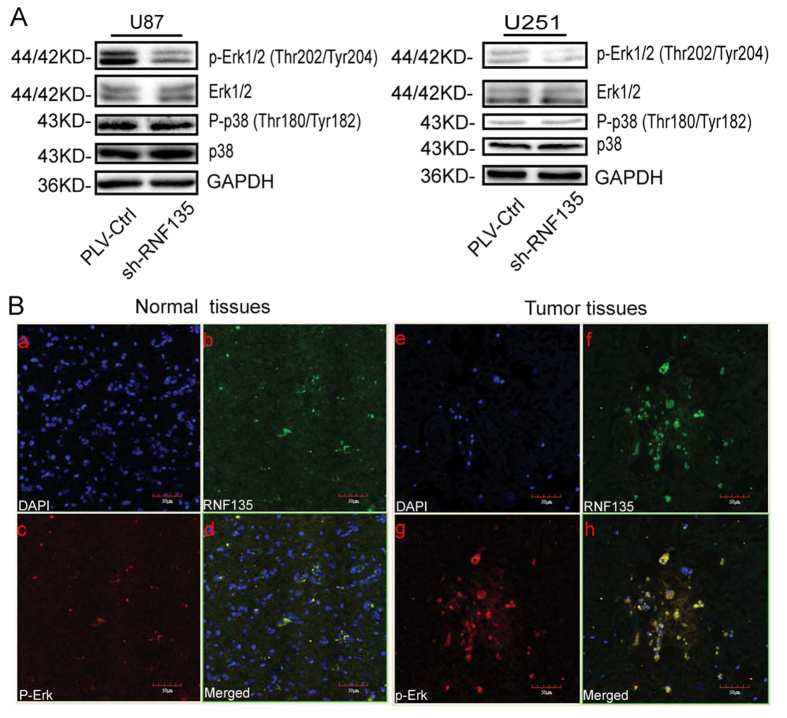
Western blotting and Immunofluorescence double labelling assay. Down-regulation of RNF135 expression attenuated the expression of P-Erk1/2, while the levels of total protein and P-p38, p38 and Erk were not affected; GAPDH was used as a loading control. Each experiment was repeated three times. (**B**): Immunofluorescence double labelling assay was used to investigate the co-expression of P-Erk and RNF135 in glioma and normal brain tissues. (**a,e**)The blue fluorescence counterstained by DAPI was in the nucleus; (**b,f**): the green fluorescence-labelled RNF135 also appeared in cytoplasm; (**c–g**): the red fluorescence-labelled P-Erk was localized in the cytoplasm; (**d,h**): yellow fluorescence appeared in the position at which the red and green fluorescence occurred simultaneously (Original magnification: 400×).

According to previous studies that the ERK and p38 pathways play an important role in modulating the cell cycle, we further investigated the effect of RNF135 on the ERK and p38 pathways. The results revealed that decreased RNF135 significantly inhibited the activation of P-Erk. However, RNF135 had no effects on the expression levels of P-p38 or the total protein levels of Erk and p38 (Fig. 7A). This work indicated that RNF135 could modulate the Erk pathway in glioblastoma. We examined the degree of co-expression of RNF135 and P-Erk in glioblastoma samples and NB tissues with fluorescent double-labelling (Fig. 7B). We observed that the red fluorescence-labelled P-Erk was localized in the cytoplasm; the green fluorescence-labelled RNF135 also appeared in the cytoplasm; the blue fluorescence counterstained by DAPI was in the nucleus. Next, we used the picture analysis software to construct the 3 fluorescence patterns; yellow fluorescence appeared in the position at which the red and green fluorescence occurred simultaneously. This finding illustrated that RNF135 and P-Erk were co-localized in the cytoplasm. Furthermore, this assay confirmed that RNF135 and P-Erk were up-expressed compared with NB tissues.

### Knockdown of RNF135 suppresses cell tumourigenicity *in vivo*

**Figure 8:**
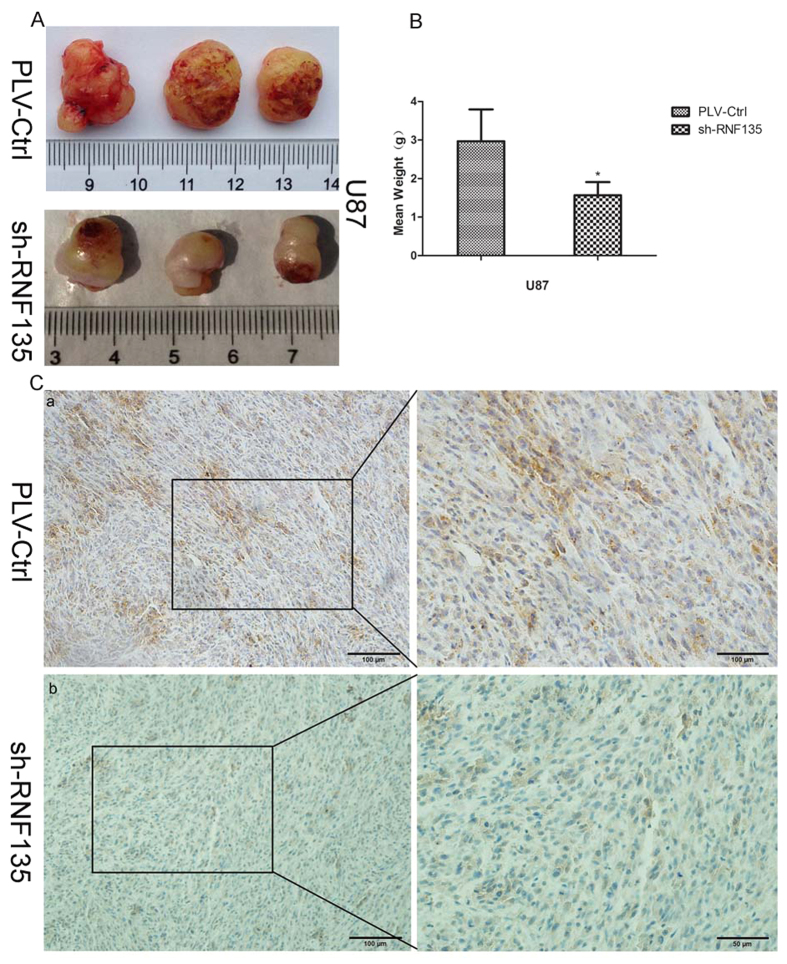
Tumour weights from subcutaneously injected PLV-Ctrl-U87 and sh-RNF135-U87 measured at 25 days post injection. (**A,B**): The tumourigenicity of sh-RNF135-U87 cells was markedly reduced *in vivo* (*P < 0.05) compared with PLV-Ctrl-U87. Ten mice were used for each treatment. (**C**): IHC staining of RNF135 expression in subcutaneous tumours of mice injected with PLV-Ctrl cells (Fig. 8C a) and shRNF135 (Fig. 8C b). (Original magnification: 400×, 100×).

To confirm the growth-increasing effects of RNF135, a nude mouse xenograft model was established by implanting shRNF135-U87 cells. The average weights of the tumours removed from mice injected with shRNF135 U87 cells and shPLV-Ctr U87 cells were 1.564 g and 2.970 g, respectively (P < 0.05) (Fig. 8A,B). Moreover, the results of immunohistochemistry staining implied that the expression of RNF135 in the shRNF135-xenografted tumours was reduced compared with the normal expression of RNF135 in shPLV-Ctr-xenografted tumours (Fig. 8C).

## Discussion

GBM is the most malignant primary brain tumour in adults. Classified as grade IV by the World Health Organization (WHO), GBM is known as the most frequent, devastating and malignant astrocytic glioma^20^,^21^. Recently, the main treatment of GBM has included surgical resection, chemotherapy and radiotherapy. Despite these very aggressive methods, the prognosis is still unfavourable. The median survival is less than 15 months, and the 5-year overall survival is below 2% for inevitable tumour reappearance^22^. Recently, researchers have started their investigation of molecular biological technologies to develop new approaches.

E3 ubiquitin-protein isopeptide ligases have been identified as bio-markers and therapy targets of glioblastoma. RNF135 is a RING domain-containing E3 ubiquitin ligase. The RNF135 protein comprises an N-terminal RING finger domain, and C-terminal SPRY and PRY motifs. The gene RNF135, which has been identified by the genome project previously, was found to be a cause of human genetic diseases such as neurofibromatosis^15^. Researchers have hypothesized that a change in the dosage of RNF135 may contribute to RNF135-involved human disease^23^.

It was reported that RNF135 haploinsufficiency leads to an overgrowth syndrome as well as learning disabilities in human beings^17^,^24^. In the present study, we first investigated the RNF135 effect in glioblastoma. First, we confirmed that the RNF135 mRNA was highly expressed in 28 glioblastoma samples compared with 12 normal brain tissues using quantitative real-time PCR. The same applied to the protein expression level. Additionally, the IHC staining assay revealed that RNF135 was highly expressed in 66.9% of glioma patients and up-expressed in 25% of normal tissues. Furthermore, the RNF135 expression levels were correlated with the grade of glioma (Table 2), and the multivariate analysis showed that the RNF135 expression level was the most important factor predicting the overall survival in patients with glioma (Table 3), with lower RNF135 expression correlated with a better survival than higher RNF135 expression (Fig. 2).

Because it contains a ring finger and PY motif, RNF135 can bind numerous proteins, indicating its breadth of functions, such as involvement with the EGFR and TGF-β^25^. Many ring finger proteins belong to the family of ubiquitin ligases, which promote the ubiquitination of proteins. This process plays a very important role in a wide spectrum of biological processes involved in development and disease pathogenesis^26^. Previous research has shown that RNF149 attenuates the increase in cell growth induced by wild-type BRAF^14^. It has been confirmed that Ring finger protein 43 (RNF43) is up-expressed in colorectal cancer and mediates cancer cell proliferation^27^, and RNF43, as a novel tumour-associated antigen, is a new target for cancer immunotherapy^28^. It was also demonstrated that RING finger proteins may play a significant role in the cancer biological process. To explore the biological function of RNF135, we knocked down RNF135 expression using shRNA in U87 and U251 cell lines. Next, we conducted a series of experiments and found that knocking down RNF135 with shRNA could significantly inhibit U87 and U251 glioma cell line proliferation and migration *in vitro* compared with that in the PLV-Ctrl groups.

Previous studies have shown that RING finger proteins play important roles in the cell cycle^29^,^30^. CDKs, p27 Kip1, p18 INK4C and p21 Waf1/Cip1 are cell cycle regulatory proteins^31^. When the cell cycle is arrested at the G0/G1 phase, the cell cycle proteins CDK2 and CDK4 are decreased, while the p21Waf1/Cip1 is highly expressed^32^. In the FACS flow cytometry results, the shRNF135 U87 and U251 cells showed 71.62% and 64.74% of G0/G1 phase cells, whereas only 62.45% and 50.71% of G0/G1 phase cells were in the PLV-Ctrl cells. The data confirmed that knockdown of RNF135 could arrest the cell cycle at the G0/G1 phase in U87 and U251 cells. A previous study showed that the RNF135 deficient mice displayed no apparent abnormalities at seven month^33^.However, in our study, knockdown RNF135 with siRNA arrested cell cycle at G0/G1 stage in U87 and U251 glioma cell lines. The discrepancy may be resulted from several reasons. First, it’s well known that when a gene was knockout, a related gene maybe upregulated to compensate for the lost function. Thus, it’s possible that the function of RNF135 in proliferation was compensated by another gene in the deficient mice. Second, the glioma cell and embryonic stem cells (ESCs) grew in different microenvironment and were regulated by different signal pathways. So, RNF135 may play different roles at cell proliferation in different cells. Actually, the discrepancy between cell and animal models was not uncommon. For example, some studies showed that the TRPC6 deficient mice is normal^34^, while knockdown TRPC6 arrested cell cycle at G2/M stage in U87 and U251 glioma cell lines^35^.Moreover, the Western blot assay showed that knockdown of RNF135 could attenuate the expression of the cell cycle protein CDK4 but enhance the expression of p27 Kip1 and p21 Waf1/Cip1.

Previously, researchers have reported that the Erk and p38 pathways are involved in the regulation of cell cycle progression^36^,^37^, cell growth, proliferation and migration^38^,^39^,^40^,^41^. Moreover, it was inferred that Cbl-c, which belongs to the family of RING finger ubiquitin ligases (E3s), decreased downstream ERK activation by RETMEN2A^42^. Furthermore, MEK kinase 1 (MEKK1) demonstrated two functions, as an upstream activator of JNK and ERK with its kinase domain and as an E3 ligase with the plant homeodomain (PHD) domain resembling the RING finger domain, providing a negative effect to inhibit ERK1/2 activity^43^. If the concentration was high enough, MEKK1 could increase ERK2 and p38 activities^44^. In this study, we observed that decreased RNF135 expression significantly attenuated the activation of P-Erk, whereas P-p38 and p38 were not affected. Furthermore, we found that RNF135 and P-Erk were highly co-expressed in glioblastoma tissues compared with normal brain tissues. Thus, RNF135’s effect on tumour progression and malignancy may be via the Erk pathway.

## Conclusion

In summary, RNF135 may have significant value as a progression indicator for patients who have glioblastoma. Evidence has confirmed that attenuated RNF135 expression could lead to suppressed cell growth and migration via inactivation of the Erk pathway in U87 and U251 glioma cells.

## Materials and Methods

### Cell culture and reagents

The human glioma U87 and U251 cell lines were supplied by the Cell Bank of Shanghai Institute of Cell Biology, Chinese Academy of Sciences. Cells were cultured in DMEM (Cat: SH30022.01B; Thermo, US) containing 10% FBS (lot: 1616964, Life Technologies, USA) at 37 °C in 5% CO_2_. MTT was from Sigma-Aldrich (St. Louis, MO; 5 mg/ml). The Erk (Cat: 7695S), P-Erk (Cat: 4370S, Cat: 13148), P-p38 (Cat: 4511s), and anti-rabbit IgG (Cat: 7074P2) secondary antibodies as well as cell cycle regulation protein antibodies CDK4 (Cat: 12790P), p27^Kip1^ (Cat: 3686p) and p21^Waf1/Cip1^ (Cat: 2947p), were all purchased from Cell Signaling Technology, Inc. (MA, USA). Anti-RNF135 was purchased from Abcam (ab174195, abcam, UK) and Sigma (Cat: AV34641, sigma, USA). Anti-GAPDH (Cat: 60004-1-lg, US) was purchased from Proteintech. All of the other reagents were of analytic grade.

### Patients and tissue samples

A total of 28 glioblastoma (WHO IV) tissues and 12 normal brain tissues from the temporal or frontal lobes of 12 brain trauma patients were obtained from the neurosurgery department of the First Affiliated Hospital of Dalian Medical University. Informed agreement was obtained from all of the patients. There were 142 glioma archived paraffin-embedded samples (including 14 astrocytic tumours, 17 oligodendroglioma, 66 anaplastic astrocytoma and 45 glioblastoma), and surgeries were performed between 2004 and 2010 in the neurosurgery department of the First Affiliated Hospital of Dalian Medical University. The median age of these 142 glioma patients were 49 years (range, 5–81 years). This group included 56 female and 86 male patients. There were 100 patients accepted the radiotherapy and chemotherapy (including 9 astrocytic tumours, 11 oligodendroglioma, 47 anaplastic astrocytoma, 33 glioblastoma) after surgical operation. Whereas, there are 42 patients didn’t accept the radiotherapy and chemotherapy (including 5 astrocytic tumours, 6 oligodendroglioma, 19 anaplastic astrocytoma, 12 glioblastoma). This work was performed with the ethical approval of the Human Ethics Committee of The First Affiliated Hospital of Dalian Medical University (approval number: KY2014–47) in accordance with institutional medical requirements, and written informed consent was obtained from each of the enrolled subjects.

### Animals

Experimental procedures and animal care were carried out according with NIH Guidelines for the Care and Use of Laboratory Animals and mice protocols were approved by the Ethics Committee of The First Affiliated Hospital of Dalian Medical University in China. Four- to six-week-old female nude mice (BALB/c-nu/nu) were obtained and raised at the Center of Experimental Animals, Dalian Medical University (China).

### Cell proliferation assay

The MTT assay was performed to assess the proliferation of cells. U87 and U251 cells were seed at a certain density (1 × 104 cells per well) in 96-well culture plates, and cultured in media for 1–6 days. Next, 10 μl of MTT reagent (5 mg/ml) was added to each well, and the plates were then incubated at 7 °C for 4 h. The formative formazan crystals were dissolved by adding 100 μl of SDS-isobutanol-HCl solution (10% SDS, 5% isobutanol, and 12 μM HCl) overnight at 37 °C. The absorbance was measured at 490 nm with a microplate reader (Bio-Rad, San Diego, CA, USA).

### Flow cytometry analysis of the cell cycle

Cells were harvested and seeded in 6-well plates. After 24 h of incubation, cells were digested and collected, following by centrifugation at 1000 rpm for 5 min at 4 °C. The deposited cells were then suspended in ice-cold PBS and washed, and then re-suspended with 70% ethanol for 30 min at 4 °C. Next, the cells were washed and re-suspended in 100 mL of PBS containing the final concentration of 50 mg/mL RNase A (Sigma, US) and 0.25% Triton X-100 for 30 min at 4 °C. Finally, the cells were stained with 10 mg/mL propidium iodide (PI, lot: 1685935, life technologies, USA) for 30 min. Next, the cells were immediately analysed in a FACScan flow cytometer (Becton Dickinson, CA, USA). All of the works were assayed three times, and the fraction of each cell cycle phase was measured.

### Immunohistochemistry

Glioma and NB tissues were cut into parts, removed into tubes and fixed in 10% formaldehyde. Tissue sections were embedded in paraffin. Pure xylene was used to dewax paraffin sections by soaking sections into pure xylene. Tissue sections were then re-hydrated in a series of water-diluted ethanol solutions. The re-hydrated tissues were blocked in serum and incubated with rabbit anti-human RNF135 antibody at 4 °C overnight. Tissues were washed twice and then incubated in biotin-labelled goat anti-rabbit antibody for 10 min at room temperature. After incubation with horseradish peroxidase (HRP), 3,3-diaminobenzidine (DAB, Cat: 15111A03, Zhong Shan Jin Qiao, China) was used to perform the peroxidase reaction. Haematoxylin was used to stain the samples, which were then counted under a microscope (Original magnification: 400×, 100×).

### Evaluation of staining

Tissue sections stained by immunohistochemistry were analysed by two pathologists blinded to the clinical parameters. Observation of RNF135 expression in the cytoplasm was performed separately. In the cytoplasm, the staining score was determined based on the cytoplasm staining intensity together with the number of positive cytoplasm-staining cells. The obtained percentage of positive staining in the cytoplasm was defined as follows: 0: <10%, 1: 10–25%, 2: 26–75%, and 3: >76%. A final staining score of 0–1 in the cytoplasm was considered low expression of RNF135. By contrast, a final staining score of 2–3 in the cytoplasm was considered high expression of RNF135.

### Immunofluorescence double labelling method

To investigate the glioma samples and NB tissues expressing the phosphorylated ERK (Thr202/Tyr204) and RNF135, a double-labelling procedure using a rabbit polyclonal antibody was carried out as described previously^45^,^46^. The antibodies included anti-RNF135, anti-P-Erk, Goat anti rabbi lgG antibody and goat anti mouse lgG antibody (Original magnification: 400×, 100×).

### Establishment of U87 and U251 cell lines with stable low expression of RNF135 short hairpin RNA

The pLVTHM-GFP lentiviral RNAi expression system was used to perform the lentiviruses expressing human RNF135 short hairpin RNA (TOP: GATCCGCAGGCCCTGTCTTCTGGAAAGCATTTTCAAGAGAAATGCTTTCCAGAAGACAGGGCCTGTTTTTTC; BOTTOM: AATTGAAAAAACAGGCCCTGTCTTCTGGAAAGCATTTCTCTTGAAAATGCTTTCCAGAAGACAGGGCCTGCG). Cells were infected with lentiviral particles containing specific or negative vectors. The selected polyclonal cells with GFP signals were analysed using FACS flow cytometry. The transfection efficiency of RNF135 was confirmed by quantitative real-time PCR analysis and Western blot assay.

### Wound Healing Assay

Cell migration and proliferation were analysed by wound-healing activity assays *in vitro*. The stably shRNA-transfected U87 and U251 cells were harvested and cultured in 6-well plates. When the cells became confluent, a cell-free scratched gap approximately 500 μm in width was created with a pipette tip in the wells of the plates, and then the cells were incubated for another 6 h and 12 h. We compared the length of the healed wound with the length of the initial wound. The lengths from two different wounded regions were measured with Image J (public domain software, http://rsb.info.nih.gov/ij/) (Original magnification: 100×).

### Boyden chamber migration assay

To perform the transwell migration assay, 1 × 10^5^ cells/ml of U87 and U251 cells were collected and diluted in FBS-free DMEM medium. Next, 100 μl of cells were seeded into the Boyden chamber (0.8 μm; Corning, USA) in 24-well plates, and 600 μl of DMEM containing 10% FBS medium was added to the wells outside the chamber. After 6 h of incubation at 37 °C, cells were washed twice with PBS. The top surface cells of the transwell were removed, and the lower surface cells were fixed with methanol. The fixed cells were stained with Giemsa solution and counted stained cells under a microscope using Image J software (public domain software, http://rsb.info.nih.gov/ij/) (Original magnification: 100×).

### Tumourigenesis in nude mice *in vivo*

U87 cells were harvested and diluted to the concentration of 1 × 10^8^ cells/ml. Next, 200 μl of cells at the density of 1 × 10^8^ cells/ml were implanted s.c. under the right shoulder of 4- to 6-week-old female BALB/c nu/nu mice. The mice were randomly divided into two groups (ten mice per group) and were administered an intratumoural injection of sh-RPS15A-U87 cells (1 × 10^7^ pfu) or sh-PLV-Ctl-U87 cells (1 × 10^7^ pfu). After 25 days of breeding, all of the mice were sacrificed, respectively, and the tumour tissues were excised and weighed. All of the animal experiments were conducted according to the principles and procedures of the National Institutes of Health Guide.

### Quantitative Real-Time PCR

According to the protocol reported previously^10^, RNA was extracted from glioma and NB tissues, as well as U87 cells, using the RNA isoPlus^®^ Reagent Kit (Cat: RR820A, Takara Biotechnology) according to the manufacturers’ protocols. The PrimeScript^®^ RT Reagent Kit (Cat: RR037A, Takara, Shiga, Japan) was used to reverse transcribe RNA into cDNA. The cDNA was amplified using the SYBR^®^ Premix Ex Taq™ Kit under 7500 Real-Time PCR System (Applied Biosystems, 7500 Real Time PCR System, Thermo, US). The cycling conditions were as follows: 40 cycles of 95 °C for 30 s, 95 °C for 5 s, 60 °C for 34 s. The comparative ΔΔCt method was used to analyse the data, and GAPDH was used as the loading control for the target genes. The primers were as follows: RNF135 (Forward: 5′-TACTGGGAAGTGGACACTAGGAATT-3′, reverse: 5′-CTTGACCATGTGCCATGCA-3′); GAPDH (Forward: 5′-GCACCGTCAAGGCTGAGAAC-3′, reverse: 5′-TGGTGAAGACGCCAGTGGA-3′).

### Western blot analysis

Tissues and cell total proteins were collected using RIPA lysis buffer containing phosphatase and protease inhibitors (Roche, Swiss) in accordance with the manufacturer’s instructions. The preparation of tissues for Western blotting was reported as described previously^11^. Glioma and NB tissues were homogenized first and then lysed in lysis buffer with shaking at 4 °C for 30 min. Proteins were quantified by the BCA protein assay agent (Cat: PC0020, Solarbio, China). Proteins were separated by SDS-PAGE, and then transferred to PVDF membranes (Cat: IPVH00010, EMD Millipore Corporation Billerica Ma01821, USA), blocked with 5% fat-free dry milk or 5% BSA in TBST and immunoblotted with primary antibodies at 4 °C overnight. The following day, the membranes were incubated with second antibodies at room temperature for 2 h. The detection of proteins on the membranes was determined using the enhanced ECL reagent by the Bio-Spectrum Gel Imaging System with image lab software (UVP, USA). Protein expression was quantified by Gel-Pro Analyzer 3.0 software.

### Statistical analysis

The qualifying data obtained were represented as the mean ± SD of at least three independent experiments. SPSS 17.0 software (SPSS, Chicago, USA) and Graph Pad Prism 5.0 (www.graphpad.com, USA) were used for statistical analysis. One-way ANOVA test or two-tailed Student’s t-test were used for comparisons between groups. Chi-squared test or Fisher’s exact test was used to analyse the difference between classified variables. Kaplan-Meier analysis and the log-rank test were used for Survival analysis. The relationship between variables and patient survival was performed using univariate and multivariate Cox proportional hazards models. P values less than 0.05 were considered to be statistically significant.

## Additional Information

**How to cite this article**: Liu, Y. *et al.* RNF135, RING finger protein, promotes the proliferation of human glioblastoma cells *in vivo* and *in vitro* via the ERK pathway. *Sci. Rep.* **6**, 20642; doi: 10.1038/srep20642 (2016).

## Figures and Tables

**Table 1:** RNF135 protein expression levels in glioma and normal brain (NB) tissues.

Group	Cases	RNF135 expression level		P
		High	Low	
NB	12	3 (25%)	9 (75%)	
Glioma	142	95 (66.9%)	47 (33.1%)	0.009

**Table 2:** Correlation between RNF135 relative expression levels and clinicopathologic characteristics in glioma patients.

Characteristics	Cases	RNF135 (%) expression level		P
		High	Low	
Age				
≥50	70	41 (58.6%)	29 (41.4%)	
<50	72	54 (75%)	18 (25%)	0.050
Gender				
Male	86	58 (67.4%)	28 (33.6%)	
Female	56	37 (66.1%)	19 (33.9%)	0.865
Histologic type				
Astrocytic tumours	14	3 (21.4%)	11 (78.6%)	
Oligodendroglioma	17	8 (47.1%)	9 (52.9%)	
Anaplastic astrocytoma	66	46 (69.7%)	20 (30.3%)	
Glioblastoma	45	38 (84.4%)	7 (15.6%)	0.000
WHO Grade				
I + II	31	11 (35.5%)	20 (64.5%)	
III + IV	111	84 (75.7%)	27 (24.3%)	0.000

**Table 3:** Summary of univariate and multivariate Cox regression analysis for overall survival duration in glioma patients.

	Univariate analysis			Multivariate analysis		
	P	HR	95% CI	P	HR	95% CI
Age (y)						
<50 vs. ≥50	0.004	1.877	1.221–2.886	0.120	1.427	0.912–2.234
Gender						
Male vs. female	0.114	0.7	0.45–1.089	0.773	1.073	0.664–1.736
Histologic type						
At. vs. Ot. vs. AAt. vs. GBM	0.000	1.757	1.361–2.268	0.148	1.307	0.909–1.879
I + II vs III + IV	0.001	7.453	2.345–23.4689	0.149	2.740	0.697–10.768
CR	0.199	0.742	0.47–1.171	0.020	0.573	0.358–0.917
RNF135 expression						
Low vs. High	0.000	3.890	2.211–6.844	0.000	2.958	1.664–5.353

(AT: Astrocytic tumours; OT: Oligodendroglioma; AAT: Anaplastic astrocytoma; GBM: Glioblastoma multiforme; CR: chemotherapy and radiotherapy).
